# Cost-Effectiveness of Procedures for Treatment of Ostium Secundum Atrial Septal Defects Occlusion Comparing Conventional Surgery and Septal Percutaneous Implant

**DOI:** 10.1371/journal.pone.0108966

**Published:** 2014-10-10

**Authors:** Márcia Gisele Santos da Costa, Marisa da Silva Santos, Flávia Mori Sarti, Kátia Marie Simões e. Senna, Bernardo Rangel Tura, Marcelo Correia Goulart

**Affiliations:** 1 Centre of Technology Assessment in Health, National Institute of Cardiology, Rio de Janeiro, Brazil; 2 University of São Paulo, São Paulo, Brazil; Groningen Research Institute of Pharmacy, Netherlands

## Abstract

**Objectives:**

The study performs a cost-effectiveness analysis of procedures for atrial septal defects occlusion, comparing conventional surgery to septal percutaneous implant.

**Methods:**

A model of analytical decision was structured with symmetric branches to estimate cost-effectiveness ratio between the procedures. The decision tree model was based on evidences gathered through meta-analysis of literature, and validated by a panel of specialists. The lower number of surgical procedures performed for atrial septal defects occlusion at each branch was considered as the effectiveness outcome. Direct medical costs and probabilities for each event were inserted in the model using data available from Brazilian public sector database system and information extracted from the literature review, using micro-costing technique. Sensitivity analysis included price variations of percutaneous implant.

**Results:**

The results obtained from the decision model demonstrated that the percutaneous implant was more cost effective in cost-effectiveness analysis at a cost of US$8,936.34 with a reduction in the probability of surgery occurrence in 93% of the cases. Probability of atrial septal communication occlusion and cost of the implant are the determinant factors of cost-effectiveness ratio.

**Conclusions:**

The proposal of a decision model seeks to fill a void in the academic literature. The decision model proposed includes the outcomes that present major impact in relation to the overall costs of the procedure. The atrial septal defects occlusion using percutaneous implant reduces the physical and psychological distress to the patients in relation to the conventional surgery, which represent intangible costs in the context of economic evaluation.

## Introduction

Atrial septal defects (ASD) are one of the most frequent congenital malformations, representing approximately 5% to 10% of heart congenital defects. Most of small atrial septal defects (less than 8 mm) close spontaneously during infancy or early childhood. Usually predominant among women, it has four types of anatomically different forms: ostium secundum (OS), ostium primum (OP), sinus venosus defect (SV) and coronary sinus defect (CS). OS defects represent around 75% of atrial septal defects [1].

During the last decades, percutaneous treatment have been considered the main treatment for several types of congenital cardiopathy, being preferred over surgical treatments due to superior results [2]. Nevertheless, the conventional surgery is usually the standard treatment in the Brazilian health system, especially in public sector hospitals, due to the price of the implant and absence of cost-effectiveness analysis that may support the adoption of the new technology.

The decision of ASD treatment is based on clinical and echocardiographic data, including signs and symptoms of right heart failure, size and location of defects, magnitude and haemodynamical impacts of left-to-right shunt, and presence and degree of pulmonary hypertension. Elective closure is recommended for ASD with echocardiographic evidences of right ventricular overcharge or shunt Qp/Qs higher than 1.5 clinically significant [3]–[5].

According to literature revision, the percutaneous implant occlusion is considered a safe and effective traditional surgical occlusion procedure, which includes several advantages, such as better aesthetical results, minor trauma, no need for extracorporeal circulation, and reduced length of stay. Nevertheless, the technique has been associated to occasional complications: device embolization, residual shunt, vascular lesion and cardiac perforation [6].

Complications may derive from unfavorable defect anatomy and wrong dimensioning of the device. The post-procedure phase may include events as device displacement, embolism and arrhythmia. Displacement or prolapsed devices have been described related to the utilization of oversized occluder or unfavorable defect anatomy, causing erosion of surrounding structures, especially aortic root wall [7].

The objective of the paper was to produce a cost-effectiveness analysis comparing two techniques of occlusion of atrial septal defects *ostium secundum*: conventional surgery and septal percutaneous implant occlusion, using the perspective of the Brazilian public health system, the Unified Health System (SUS). The analysis of cost-effectiveness for evaluation of both techniques considered the following outcomes: mortality, immediate ASD occlusion, occlusion during follow-up, length of stay, need for repetition of the procedure, and complications.

The group of ASD patients treated by conventional surgery was identified as “control” and the group of ASD patients treated using the septal percutaneous implant occlusion technique was identified as “intervention” for the evaluation purposes of the manuscript, as the conventional surgery remains the standard protocol in most public hospitals of the Brazilian health system due to absence of reimbursement of the implant costs by the government. Since approximately 75% of the Brazilian population relies on the public sector coverage for health care, the economic evaluation of the techniques performed in a reference hospital may provide evidences to support further analysis for adoption and reimbursement by the Brazilian Ministry of Health.

## Materials and Methods

### Ethical considerations

The project was submitted to Brazil Platform (CAAE: 09842112.3.0000.5272), assessed and approved unanimously by the Research Ethics Committee of the National Institute of Cardiology, where data collection was performed. Opinion number: 155 223. Conclusion date: Nov. 27, 2012.

The atrial septal defect (ASD) occlusion is traditionally performed through cardiac surgery, and the access to cardiac area is obtained through classic median sternotomy with extracorporeal circulation, which involves a post-operation period in intensive care unit, a requirement that contributes to significant increase of costs in the Brazilian health system [8].

The occlusion using percutaneous implant is performed with general anesthesia in patients that present atrial septal defect in adequate size in relation to its diameter, measured under fluoroscopy and guided by transesophageal echocardiogram. The device is deployed when in stable adequate position in relation to the defect. The patient is heparinized during 24 hours and usually discharged in the next day.

The ideal ASD for percutaneous occlusion presents firm margins, and adequate dimensions for mitral valve, superior and inferior vena cava, aortic root, coronary sinus, and tricuspid valve to accommodate the device, with at least 20 mm diameter. In practice, however, ASD occlusion is performed in most cases that present diameter less than 40 mm and margin greater than 5 mm from the mitral valve, and vena cava and right superior pulmonary vein ports. A smaller margin for the coronary sinus (2–3 mm) is acceptable to accommodate some device overlap [3].

A model of analytical decision was structured with symmetric branches in order to estimate the cost-effectiveness ratio between the conventional surgery and septal percutaneous implant occlusion, using the software TreeAge 2012 Pro.

In order to assemble the decision tree model, a systematic review of literature was performed in Medline, Trip Database e Lilacs databases, including any types of study design (cohort, clinical trials, case studies, and reviews) involving procedures for the treatment of atrial septal defects *ostium secundum* performed in adult and infant individuals, including patients from 0 to 21 years-old published in English, Portuguese and Spanish during the last 10 years until August, 2012.

The literature search strategy for systematic review of ASD occlusion procedures included combinations of specific terms of interest in each bibliographic database. In the case of Medline, the search strategy obtained as result 284 titles. In the case of Trip Database, the search strategy obtained just one title, which was not considered appropriate for the review. In the case of Lilacs, the search strategy resulted in three titles, none of which was considered appropriate for the systematic review (Table 1).

**Table 1:** Elements used in the literature search strategy for systematic review of ASD occlusion procedures.

Database	Searchstrategy	Titles	Abstracts	Papersselected
Medline	((“SeptalOccluder Device”[mh] OR amplatzer[tw] OR ((occluder*[tw] ORclosure*[tw]) AND device*[tw])) AND (surger*[tw] OR surgic*[tw] ORatrioseptoplast*[tw] OR “Heart Septal Defects, Atrial/surgery”[mh]) AND(“Heart Septal Defects, Atrial”[mh] NOT (“Foramen Ovale, Patent”[mh] NOT(“Heart Septal Defects, Atrial”[mh] NOT “Foramen Ovale, Patent”[mh]))))AND (Therapy/Broad[filter] OR Prognosis/Broad[filter])	284	284	68
TripDatabase	“Heart Septal Defects, Atrial” AND “Prostheses and Implants”	01 (CDR)	0	0
LILACS	Interatrial communication [Words] and Prostheses and Implants [Words] and Congenital cardiopathy [Words]	03	0	0

**Figure 1:**
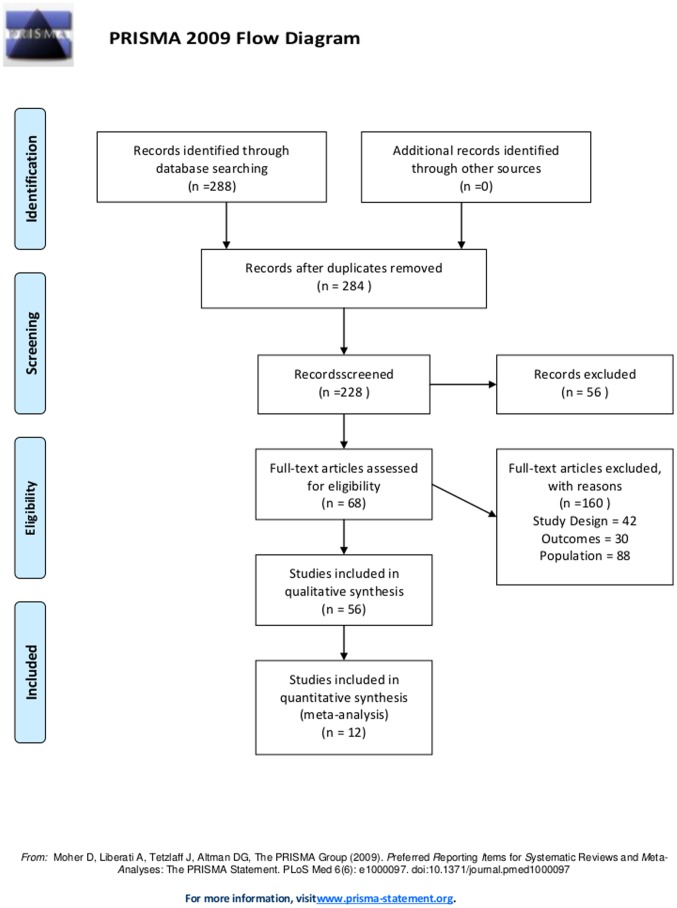
Flow diagram of the decision model for ASD occlusion procedures.Brazil, 2012.

The selection of papers was performed by four experts distributed in two groups; each group received 142 manuscripts (of the 284 titles initially located) and independently indicated the studies appropriate for the systematic review. A fifth expert was responsible for tiebreaking. The inclusion criteria were: comparison of the two treatment techniques (conventional surgery and percutaneous occluder implant), analysis of outcomes related to efficacy and safety of both treatment techniques in the short, medium and long term, and treatment of patients diagnosed with atrial septal defects *ostium secundum* (Figure 1).

Following primary analysis of the abstracts, 68 papers were selected for systematic review, and 12 studies [9]–[20] concerning the main outcomes for ASD occlusion procedures were used to perform a meta-analysis of evidences regarding conventional surgery and septal percutaneous implant occlusion (Table 2).

**Table 2:** Summary of the articles included in the meta-analysis, 2012.

*Authors*	*Title*	*Journal*	*Year of publication*	*Type of study*
Du ZD, Hijazi ZM, Kleinman CS, et al.	Comparison between transcatheter and surgical closure of secundum atrial septal defect in children and adults: Results of a multicenter non randomized trial	J Am Coll Cardiol	2002	Non randomized multicenter clinical trial
		39(11):1836–44		Children and adults
				Percutaneous implant (n = 442)
				Conventional surgery (n = 154)
				Follow-up 12 months
Durongpisitkul K, Soongswang J, Laohaprasitiporn D, et al.	Comparison of atrial septal defect closure using amplatzer septal occluder with surgery	Pediatr Cardiol	2002	Non randomized clinical trial
		23(1):36–40		Children and adults (2y–69y)
				Percutaneous implant (n = 35)
				Conventional surgery (n = 64)
				Follow-up 12 months
Kong X, Cao K, Yang R, et al.	Transcatheter closure of secundum atrial septal defect using an Amplatzer septal occluder	Chin Med J (Engl)	2002	Cohort
		115(1):126–8		30 children and adults (5y–55y)
				Follow-up 3months
Bettencourt N, Salomé N, Carneiro F, et al.	Atrial septal closure in adults: Surgery versus Amplatzer - comparison of results	Rev Port Cardiol	2003	Retrospective cohort
		22(10):1203–11		63 adultos (13y–72y)
				Percutaneous implant (n = 38)
				Conventional surgery (n = 25)
				Follow-up 12 months
Hessling G, Hyca S, Brockmeier K, et al.	Cardiac dysrhythmias in pediatric patients before and 1 year after transcatheter closure of atrial septal defects using the amplatzer septal occluder	Pediatr Cardiol	2003	Cohort
		24(3):259–62		23 children (1.8y–15y; average 7.1y)
				Follow-up 12 months
Kannan BR, Francis E, Sivakumar K, et al.	Transcatheter closure of very large (>or = 25 mm) atrial septal defects using the Amplatzer septal occluder	Catheter Cardiovasc Interv	2003	Retrospective cohort
		59(4):522–7		45 patients (34y +/− 13y)
				Follow-up 3to 30 months
Bialkowski J, Karwot B, Szkutnik M, et al.	Closure of atrial septal defects in children: Surgery versus Amplatzer device implantation	Tex Heart Inst J	2004	Clinicaltrial
		31(3):220–3		91 children
				Percutaneous implant (n = 47)
				Conventional surgery (n = 44)
				Follow-up 3.9y +/− 0.9y
Braga SL, Sousa AG, Pedra CA, et al.	[Clinical efficacy and safety of the percutaneous treatment of secundum atrial septal defect with the Amplatzer occluder]	Arq Bras Cardiol	2004	Cohort
		83(SpecNr)7–13		49 children and adults
				Follow-up 12 months
Brown SC, Bruwer AD, Harrisberg J, et al.	Percutaneous closure of interatrial defects: The Free State experience	Cardiovasc J S Afr	2004	Case study
		15(1):28–31		7children (3.7y–16.6y)
				Follow-up 12 months
Masura J, Gavora P, PodnarT	Long-term outcome of transcatheter secundum-type atrial septal defect closure using Amplatzer septal occluders	J Am Coll Cardiol	2005	Cohort
		45(4):505–7		151 children and adults
				Follow-up 3years
Vida VL, Barnoya J, O'Connell M, et al.	Surgical versus percutaneous occlusion of ostium secundum atrial septal defects: Results and cost-effective considerations in a low-income country	J Am Coll Cardiol	2006	Retrospective cohort
		47(2):326–31		111 children and adults
				Percutaneous implant (n = 83)
				Conventional surgery (n = 28)
				Follow-up 12 months
Jones TK, Latson LA, Zahn E, et al.	Results of the U.S. multicenter pivotal study of the HELEX septal occluder for percutaneous closure of secundum atrial septal defects	J Am Coll Cardiol	2007	Multicenter clinical trial
		49(22):2215–21		263 children and adults
				Percutaneous implant (n = 135)
				Conventional surgery (n = 128)
				Follow-up 12 months

The data analysis was performed using the statistical software R version 3.0.1, using two techniques. Intervention studies including outcomes expressed as discrete variables (number of outcomes registered in the intervention and control groups of patients) were analyzed by Mantel-Haenszel meta-analysis methodology; and intervention studies including outcomes expressed as continuous variables were examined with inverse variance meta-analysis methodology. Other descriptive studies focusing solely evaluation of septal occluders were used to obtain probabilities and odds-ratio (OR) of the outcomes described.

Outcomes considered for cost-effectiveness analysis were ASD occlusion, repetition of the procedure and complications. There were no statistically significant differences between the conventional surgery and the percutaneous implant regarding other potential outcomes (mortality, long term sequelae and life years gained). Statistical analysis of treatment effects considered outcomes expressed in odds ratio with 95% confidence interval using the random model.

Heterogeneity among studies was analyzed by values I^2^, confidence interval and p-values (p<0.05); and the quality of evidences obtained was categorized as low using GRADE [21].

The design of the model implied that the patients are conducted for the ASD occlusion procedure, including a probability for the occurrence of complications [9], [15], [22] and the need for repetition of the procedure or not [23]. In the case of the event of non-occlusion, an evaluation of haemodynamical repercussion determined the need for the repetition of the procedure.

The decision tree model and the effectiveness outcomes selected were validated by a Delphi panel with cardiopediatry specialists in order to ensure its accuracy in relation to the procedures analyzed. The lower number of surgical procedures performed for atrial septal defects occlusion at each branch was considered as the effectiveness outcome, since the most important result pointed out by the specialists in Delphi panel was the number of averted surgeries.

The direct medical costs of each treatment technique (conventional surgery and septal occluder implant) were estimated through micro-costing technique using data from a reference hospital in cardiology of the Brazilian public sector, considering the inputs of procedures registered in the medical records from patients diagnosed with atrial septal defects *ostium secundum* enrolled for treatment during the period from 2006 until 2011, including one year follow-up. During the period considered, 54 patients were treated using conventional surgery and 49 patients were treated using septal percutaneous implant in the public hospital.

Although the percutaneous implant is already commonly adopted for ASD treatment at several hospitals in developed countries, the conventional surgery remains the standard protocol for ASD treatment in Brazilian public hospitals. Thus, the selection of patients for treatment using each technique in the reference hospital was based on three criteria: anatomical feasibility for placement of the implant, authorization for treatment using septal percutaneous occluder (obtained from the patient or the accompanying person responsible for the patient) and availability of the implant at the hospital.

The sample of patients selected to perform the economic analysis comparing the conventional surgery to the percutaneous implant matched the first criteria, being assigned to each group according to the second and third criteria described, in order to ensure comparability of the cases.

The prices of the inputs and the wages of human resources for each treatment technique were extracted from a Brazilian public sector database for acquisition of health-related materials (System for Management of Reference Prices Table of Procedures, Medication and Orthotics, Prosthetics, and Special Materials from the Brazilian Unified Health System - SIGTAP, version 1.2.0909141204).

Costs for treatment of the complications were calculated as the average of the three complication levels (major, moderate, and minor) in relation to the costs of patients without complication, since the stratification did not impact the overall costs. A sensitivity analysis included price variations of the percutaneous implant ranging from US$6,626.21 to US$13,106.80 (values registered by the Brazilian public health system from 2007 and 2012).

The direct medical costs estimated for each treatment technique (Table 3) and the probabilities for each outcome obtained from the meta-analysis (Table 4) were inserted in the decision tree model in order to allow comparison of both conventional surgery and septal percutaneous implant.

**Table 3:** Costs related to the surgery for ASD occlusion procedures, Brazil, 2012.

Surgery Costs		
Inputs	Quantity	Total Cost (in US$)
Atrial septal occlusion(8 days of hospitalization, incl. 1–3 days of ICU)		5,782.66
Auto transfusion set	2	111.13
Catheter (central access peripheral insertion)	1	96.12
Catheter (venous central double lumen)	2	94.64
Centrifugalpump	1	354.16
Echocardiogram	8	155.11
Electrocardiogram	8	20.00
Electrode for temporary epicardial pacemaker	2	28.05
Extra corporeal circulation monitoring		29.13
Extra corporeal circulation set	2	1,681.19
Haemogram	8	15.96
Medication and other medicalsupplies		2,209.30
Organic patch	2	87.48
X ray	8	36.89
**Global cost of surgery**		**10,701.80**
**Global cost of complications**		**504.02**

Septal Percutaneous Implant		
Inputs	Quantity	Total Cost (in US$)
Transthoracic echocardiogram	1	19.39
Transesophageal echocardiogram	1	80.10
Catheter (guide for percutaneous transluminal angioplasty)	2	189.76
Steerable guidewire for angioplasty	2	189.76
Release set of interatrial prosthetics	1	1,310.68
Prosthetics for ASD occlusion	1	5,800.97
Percutaneous occlusion ductusarteriosus (1,5days of hospitalization)		382.46
Medication and other medicalsupplies		232.96
**Global cost of septal percutaneous implant**		**8,206.06**
**Global cost of complications**		**204.38**

**Table 4:** Probabilities obtained in literature review and inserted in decision model, Brazil, 2012.

Outcomes	Conventional Surgery	Septal Percutaneous Implant
Immediate ASD occlusion	99.11%	93.23%
		OR 13.78; IC 95% [6.9; 27.53]
		
Occlusion during follow-up	-	95.21%
		OR 19.88; IC 95% [15.21; 25.97]
		
Need for repetition of the procedure	0.37%	1.44%
		OR 1.62; IC 95% [0.36; 7.29]
		
Complications	12.87%	3.89%
		
Major complications*	5.24%	1.78%
		OR 0.34; 95% IC [0.18; 0.62]
		
Moderate complications	12.63%	3.41%
		OR 0.04; IC 95% [0.02; 0.08]
		
Minor complications*	22.36%	6.26%
		OR 0.28; IC 95% [0.2; 0.41]

Costs in US$	Conventional Surgery	Septal Percutaneous Implant
Procedures	10,701.80	8,206.06
		
Complications	504.02	204.38
		

(*) Data compromised due to literature heterogeneity.

## Results

**Figure 2:**
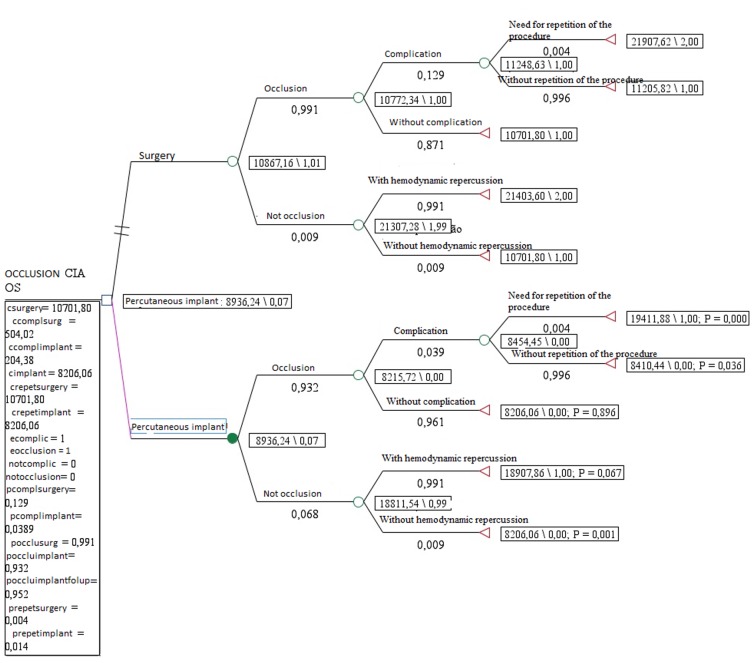
Results of the decision model for ASD occlusion procedures.Brazil, 2012.

The results obtained from the decision model demonstrated that the percutaneous implant was more cost effective at a cost of US$8,936.34 with a reduction in the probability of surgery occurrence in 93% of the cases (Figure 2), that is, presents higher effects (higher probability of averted surgeries) and a reduction of US$1,930.92 in ASD treatment costs.

**Figure 3:**
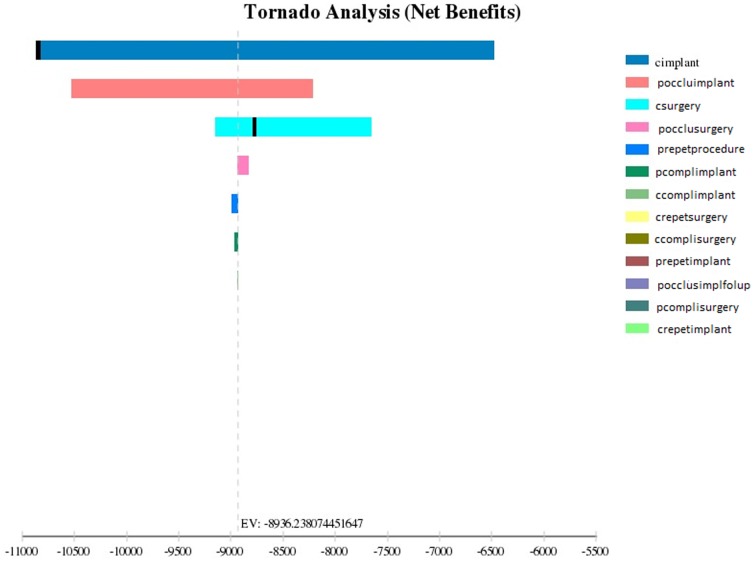
Results of tornado analysis for ASD occlusion procedures.Brazil, 2012.

The tornado analysis (Figure 3) indicates that the probability of atrial septal communication occlusion and the cost of the implant are the factors that present major influence in cost-effectiveness ratio.

**Figure 4:**
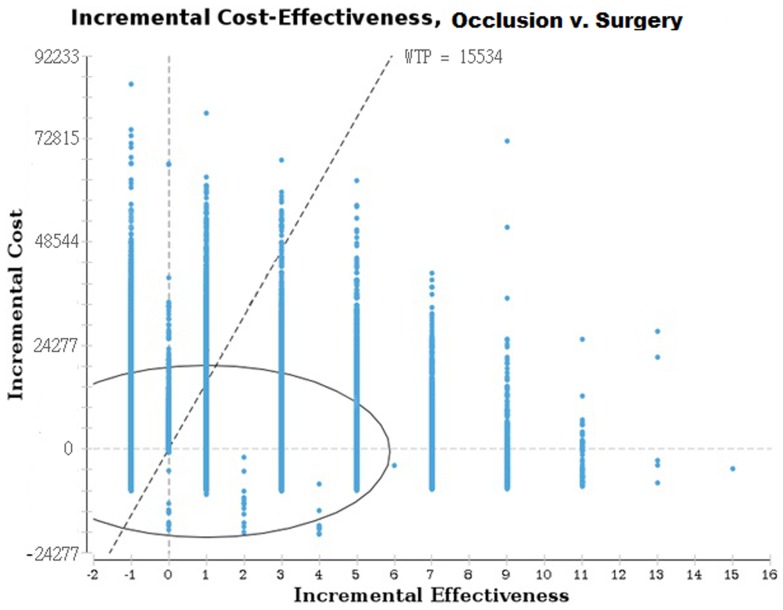
Results of incremental cost-effectiveness analysis for ASD occlusion procedures (effectiveness  =  avoided surgery).Brazil, 2012.

The incremental cost-effectiveness ratio analysis, performed using Monte Carlo simulations, demonstrates that the adoption of percutaneous implant presents favorable cost-effectiveness ratio in comparison to conventional surgery in 68.91% of the situations simulated (Figure 4).

**Figure 5:**
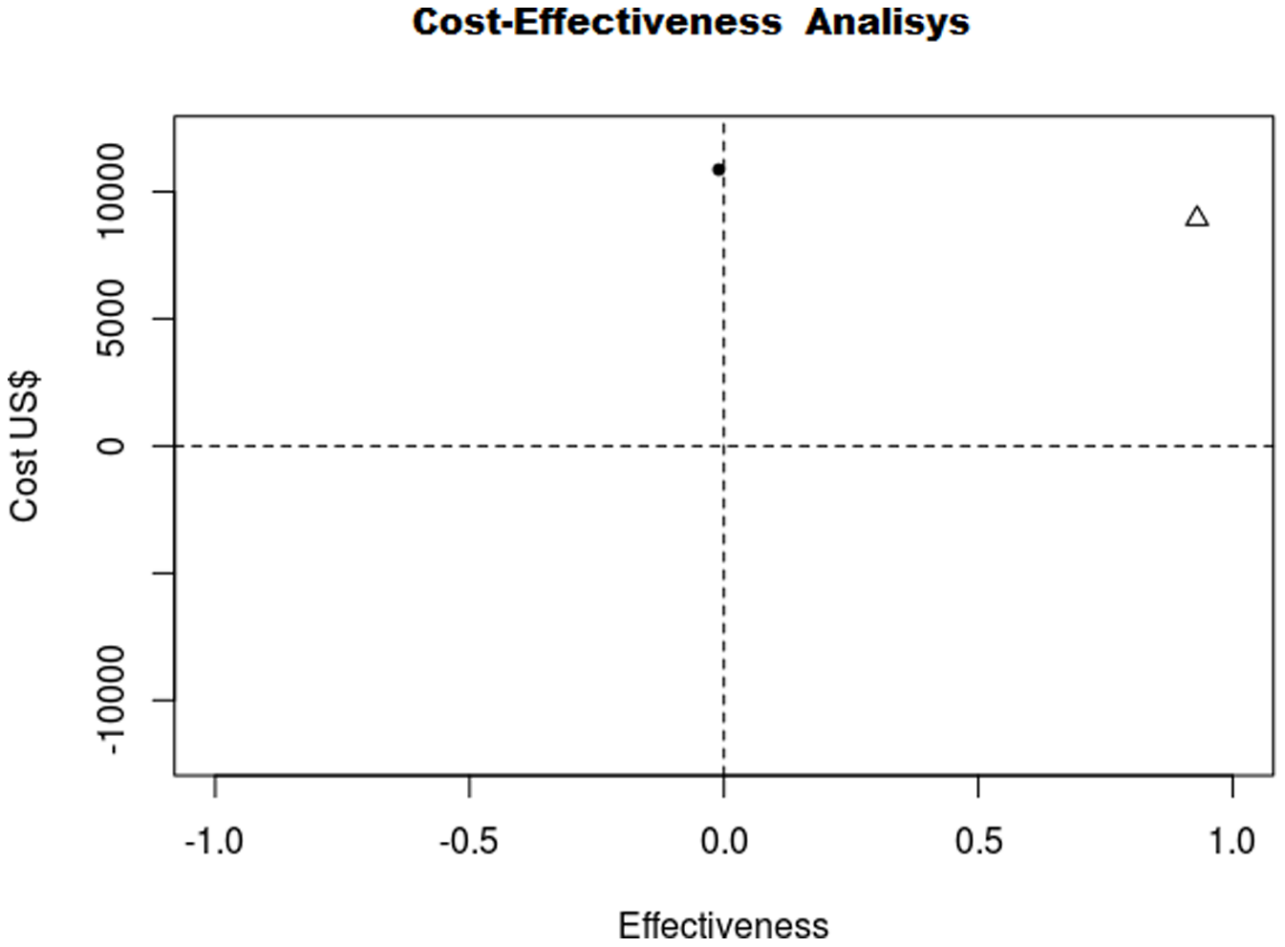
Results of cost-effectiveness analysis for ASD occlusion procedures (effectiveness  =  avoided surgery).Brazil, 2012. Δ  =  percutaneous implant/•  =  conventional surgery.

The results of cost-effectiveness analysis for ASD occlusion procedures are demonstrated graphically (Figure 5).

In the decision tree model proposed, a comparison of two technologies for atrial septal defects occlusion was performed (conventional surgery and percutaneous implant), based on the probability of occurrence of complications and the need for repetition of the procedure. In the literature review, there were no studies including outcome measures expressed into Quality-Adjusted Life Years (QALY) and there were no statistically significant differences between the techniques in relation to other outcomes (mortality, long term sequelae and life years gained).

In relation to the probability of occurrence of complications, the study analyzed three categories reported in literature review: major, moderate and minor complications. In the decision model, however, the stratification had no impacts in the costs for treatment of the complications considered; thus, the average cost was used in the study.

Regarding the possibility of ineffective closure, the haemodynamical repercussion was evaluated, determining the need for repetition of the procedure. The repetition of the procedure was defined as repetition of the complete intervention previously performed. The costs and the probabilities for occurrence of each event were estimated using meta-analysis of selected papers.

There was indication of low probability of major complications occurrence (1.78%) in the intervention group, however, data analyzed were compromised by the heterogeneity of the studies. There was a 66% reduction of incidence of major complications in the intervention group (OR 0.34; 95% IC [0.18; 0.62]).The estimated probability of moderate complications in the intervention group is 3.41% (OR 0.04; IC 95% [0.02; 0.08]); indicating a reduction of 73% in moderate adverse events in the intervention group. In the case of minor complications, the probability of occurrence in the intervention group was 6.26%, however the heterogeneity of studies also compromised the results (OR 0.07; IC 95% [0.03; 0.13]). Compared to the control group, there was a decrease of 72% in minor complications in the intervention group (OR 0.28; IC 95% [0.2; 0.41]).

The ASD occlusion after surgical procedure occurred in 99.11% cases; whereas in the case of percutaneous implant there may be complete occlusion during the follow-up period. Residual shunt may occur in both cases.

In the studies analyzed, there was a 93.23% probability of immediate complete occlusion (OR 13.78; IC 95% [6.9; 27.53]) in comparison to the control group, and a 95.21% probability of complete occlusion during follow-up (OR 19.88; IC 95% [15.21; 25.97]).

The probability of procedure repetition was favorable to the control group (OR 1.62; IC 95% [0.36; 7.29]). In the intervention group, the need for procedure repetition usually occurs due to inappropriate positioning of the device (1%to 5%) [15].

The sensitivity analysis was performed applying variations in the costs of the occluder (ranging from US$6,626.21 to US$13,106.80; being prices registered in the reference hospital from the Brazilian Ministry of Health). The percutaneous implant was the more cost effective procedure. According to the Monte Carlo simulations performed, in 68.91% of the cases the septal occlude presented favorable cost-effectiveness ratio in comparison to the conventional surgery.

## Discussion

Septal occluders are available for ASD treatment during the last three decades. King et al. performed the first percutaneous ASD occlusion in 1976, later supported by new devices and technologies, which reinforced the utilization of the technique among specialists [19].

Nevertheless, public hospitals in Brazil still adopt the conventional surgery as standard technique for ASD treatment, based on simplistic government decisions considering budget constraints in relation to the price of the percutaneous implant. There is lack of economic evaluations comparing conventional surgery and percutaneous implant techniques, which may provide information for governments in developing countries to perform further analysis on potentially cost-reducing health technologies to improve national health systems. Most studies found during the literature review showed limited methodological scope, usually focusing the description of case studies, reviews, and cohorts that compare two types of procedures, evaluation of efficacy and safety of interventions.

Vida et al. [19] compared the two procedures using cost-effectiveness analysis in Guatemala, based on the revision of medical charts from 83 patients with septal percutaneous implant and 28 patients that went through conventional surgery, indicating that septal percutaneous implant presented clinical advantages in relation to the conventional surgery at higher costs. Results comparing both procedures in relation to the complications were not statistically significant, whereas the length of stay and utilization of blood products presented differences statistically significant that resulted in advantages for septal percutaneous implant.

One important limitation of the study performed in Guatemala was the comparison of global direct costs, which did not take into account the possible variations in medication and laboratory exam prices, among other variables that may influence the results of the cost-effectiveness analysis. In spite of this, the authors conclude that the septal percutaneous implant presents cost 27% higher than the conventional surgery, considering that the cost of the device corresponds to 65% of the global costs of the procedure.

There is a limited number of cost effectiveness studies [25]–[27] on the comparison of percutaneous implants with other ASD treatment techniques, demonstrating some advantages of the percutaneous implant; however, most of the results did not present differences statistically significant among the procedures analyzed. Kim and Hijazi [27] showed substantial reduction of costs attributable to differences in length of stay and intensive care unit utilization, regarding the percutaneous implant in relation to the conventional surgery. These studies corroborate the results obtained in the research conducted in Brazil, confirming the impact of implant prices on overall ASD treatment costs.

The proposal of a decision model seeks to fill a void in the academic literature, since there were no studies including an economic analysis of ASD occlusion procedures identified until 2012. Furthermore, the results of the systematic revision performed indicated solely case studies, cohorts and clinical trials regarding the comparison of procedures for ASD occlusion. Approximately 82% of papers identified during systematic review were case studies. The meta-analysis included only the best sources of evidences from the cohorts and clinical trials selected; however, it still resulted in low quality of evidences according to GRADE.

The decision model proposed includes the outcomes that present major impact in relation to the overall costs of the procedure. Both procedures presented low mortality rate with no statistical difference in the literature (OR = 0.79 95% CI [0.18;3.5]; ×2(6) = 1.06; p = 0.9832), thus, it was not included as outcome in the model.

Post-procedure complications, immediate ASD occlusion and repetition of the procedure were the most important outcomes identified during literature review, as confirmed by meta-analysis and Delphi panel of specialists, being included in the model estimation.

The percutaneous implant present lower probability of complications, considering that it is less invasive than the conventional surgery. Therefore, the procedure using percutaneous implant also presents shorter length of stay (1.5 days of hospitalization) than the conventional surgery (8 days of hospitalization, including 1 to 3 days in intensive care unit) in the treatment for occlusion of atrial septal defects, resulting in lower costs of the intervention using the implant (US$382.46) than the surgical procedures (US$5,782.66), which compensates with several advantages the price of the device.

The immediate ASD occlusion occurs in 99.11% of cases treated by conventional surgery and 93.23% of cases using percutaneous implant (which presents 95.21% probability of complete occlusion during follow-up). In both cases, there is possibility of residual shunt.

The need for repetition of the procedure may occur just in case of significant haemodynamical repercussion due to ineffective occlusion, depending on medical decision, being reported in 1.44% of the cases involving percutaneous implant and 0.37% of the cases involving conventional surgery.

The estimation of costs of the treatment using percutaneous implant indicated cost savings of US$1,930.92 per procedure, and a reduction in the probability of surgery in 93% of the cases. The results obtained indicate that the percutaneous implant was more cost effective in this analysis; especially considering that evidences in the literature indicate that it is a safe and effective alternative for ASD occlusion in comparison to the conventional surgery, presenting excellent aesthetic results, minor trauma, no need for extracorporeal circulation, and reduced length of stay; outcomes that favor the patient's recovery.

There is no threshold values used to define a cost-effective procedure in Brazil. However, to further explore the cost-effectiveness limits, the results of the study done by the World Health Organization were used for comparison, which suggests that three times the Gross Domestic Product (GDP) [24] is a relevant indicator of cost effectiveness. Considering the lack of further data, the triple of the Brazilian Gross Domestic Product (GDP) was used as proxy threshold for the analysis (equivalent to US$15,534.00). The sensitivity analysis included positive and negative variations of 30% in implant prices. The simulations performed were based on probability distributions using the range of values in the interval, resulting in 68.91% scenarios that presented acceptable cost-effectiveness values of the intervention. Compared to conventional surgery percutaneous implantation presented an ICER of US$ -2,054.17 per surgery avoided (Table 5).

**Table 5:** Summary of cost-effectiveness analysis results, Brazil, 2012.

Treatment	Cost (US$)	Incremental cost	Effectiveness (Avoided surgery)	Incremental effectiveness	CER	ICER
Percutaneous implant	8,936.24	0	0.93	0	127,660.57	
Conventional surgery	10,867.16	−1,930.92	−0.01	0.94	11,560.81	−2,054.17 dominated

CER  =  cost-effectiveness ratio; ICER  =  incremental cost-effectiveness ratio.

Relatively to the occurrence of complications related to the procedure, Du et al. [9], Bialkowiski et al. [15], and Chessa et al. [22] describe three levels of complications (major, moderate, and minor); nonetheless, the model presented in this study includes the average probability of complications for the procedure, since the stratification did not show significant impacts in the results of the model.

The decision model indicates that the septal percutaneous implant was more effective in relation to the conventional surgery, and the sensitivity analysis demonstrates that the implant cost and the probability of occlusion are determinants of the procedure dominance.

Some limitations of the study include the lack of evidences from randomized controlled trials and meta-analysis in the literature review. Thus, GRADE [21] analysis showed that the literature evidences used in the decision model was classified as presenting low quality. However, this should not compromise significantly the results obtained, due to the consistency of the study design and availability of databases for the cost-effectiveness analysis; a consolidated technique among specialists.

There was an absence of evidences regarding outcomes related to effectiveness of the technique; therefore, other studies should be conducted in order to register additional impacts of the procedure to the patients and the national health system.

The costs of the procedures, hospital services and health professionals were estimated using government databases, which comprise the standard values, granted for public health organizations, supplemented by the estimation of costs of additional exams performed, medication and other medical supplies used during the length of stay of the patients registered from 2007 and 2010 in the hospital.

Finally, the implant price was adapted from the costs of *ductus arteriosus* percutaneous occlusion with coils release, which demonstrates the need for standardization of patterns for reimbursement of health procedures in the Brazilian public health system.

## Conclusions

The usual practice of ASD occlusion procedures using septal percutaneous implant in the hospital shows that the technique is a safe and effective alternative in relation to the conventional surgery, including several advantages, such as reduced trauma, reduced length of stay, no need of extra corporeal circulation, lower volume of blood transfusion and excellent aesthetic results.

The present study consolidates evidences on clinical results of surgical procedures available in the academic literature, and demonstrates that the ASD occlusion using septal percutaneous implant is significantly more effective and cheaper than the conventional surgery, and represents the most cost-effective procedure in comparison to the conventional surgery in the perspective of the Brazilian public health system.

## Supporting Information

Checklist S1 **PRISMA Checklist.**
(DOC)
