# Correction: Cost-Effectiveness of Procedures for Treatment of Ostium Secundum Atrial Septal Defects Occlusion Comparing Conventional Surgery and Septal Percutaneous Implant

**DOI:** 10.1371/journal.pone.0122426

**Published:** 2015-03-26

**Authors:** The PLOS ONE Staff

The last author’s name is incorrect. The correct name is: Marcelo Goulart Correia. The correct citation is: Costa MGSd, Santos MdS, Sarti FM, Senna KMSe, Tura BR, Correia MG. (2014) Cost-Effectiveness of Procedures for Treatment of Ostium Secundum Atrial Septal Defects Occlusion Comparing Conventional Surgery and Septal Percutaneous Implant. PLoS ONE 9(10): e108966. doi: 10.1371/journal.pone.0108966

In the Author Contributions section, author Marcelo Goulart Correia’s initials should be MGC. The correct contributions are: Conceived and designed the experiments: MGSC MSS. Performed the experiments: MGSC MSS KMSS MGC FMS. Analyzed the data: MGSC MSS KMSS BRT MGC. Contributed reagents/materials/analysis tools: MGSC MGC BRT. Wrote the paper: MGSC FMS. Systematic review: MGSC KMSS MSS MGC. Data collection costs: MGSC KMSS FMS.
